# Expanding the boundaries: investigating the integration of contextual information across a spectrum of inter-trial variability

**DOI:** 10.3389/fpsyg.2024.1494698

**Published:** 2024-11-20

**Authors:** Ruyi Qiu, Yanzhi Mo

**Affiliations:** Department of Psychology, Hunan University of Chinese Medicine, Changsha, China

**Keywords:** contextual stimulus, inter-trial variability, stimulus-response (S-R) episodes, binding structures, integration threshold

## Abstract

It is well-documented that feature integration across perception and action creates a retrievable episodic representation, known as a stimulus-response episode or an event file. Previous studies have demonstrated that a task-irrelevant stimulus, which functions as contextual information, can be integrated in various ways. In some cases, the context modulated the binding between a stimulus and a response, resulting in a configural binding structure. In other cases, the context was found to be directly bound with the response in a binary fashion. The current study examined the integration of context within a stimulus-response (S-R) episode, with a focus on the role of inter-trial variability. Specifically, the context variability was manipulated across five experimental groups, ranging from the minimum to the maximum level. The minimum-variability group maintained a consistent pattern of two context tones per block, while the maximum-variability group used a uniformly random order of eight different context tones. Intermediate groups progressively employed greater degrees of variability in the presentation of contextual stimuli. Results showed that the integration of context changed as a function of its variability level: The contextual stimuli with minimal to low level of variability did not exhibit a pattern of integration, while those with moderate to high variability were involved in a configural binding with another stimulus and the response. Only when the context exhibited maximal variability did it become directly bound with the response in a binary fashion. The current findings extend previous assumptions about saliency thresholds for stimulus integration into the realm of inter-trial variability and underscore the role of stimulus uncertainty in shaping context integration. Possible underlying mechanisms are discussed.

## Introduction

The environment that human beings live in is continuously changing. It is assumed that in order to deal with the intricate external world, the human brain integrates perceptual and action information into a transient episodic representation, facilitating efficient control of human behavior. The proposed integration of perceptual and action features has been intensively investigated in previous studies (see Frings et al., 2020, 2024; Hommel, 2004 for overviews), which provide strong evidence that the feature binding is a general mechanism underlying human information processing and action control.

It is further assumed that the feature binding across perception and action in a stimulus environment creates a common episodic representation, known as a *stimulus-response (S-R) episode* or an *event file*. Once established, the S-R episode can be retrieved upon feature repetition, a process that leads to either facilitation or impairment of performance, depending on whether the retrieved information is compatible with the current processing requirement or not. The so-called stimulus-response binding and retrieval processes have been observed not only with task-relevant stimuli, namely, the target (e.g., Hommel, 1998) and the distractor (e.g., Frings et al., 2007), but also with task-irrelevant elements like contextual features (e.g., the form of a stimulus when its color should be responded to, Hommel, 1998), and contextual stimuli, which we here defined as any additional (i.e., task-irrelevant) stimuli accompanying the target and the distractor without being assigned to any response throughout the experiment (Mayr et al., 2018; Qiu et al., 2022a,b).

Interestingly, the context has been reported to be integrated in various ways. In some instances, a unitary binding structure that links the contextual stimulus and the response was observed (Qiu et al., 2022a,b). Involving only two elements, this type of binding is referred to as a *binary binding* and was commonly found between a task-relevant stimulus and a response (Hommel, 2004). In other instances, a contextual modulation of the binary binding between a task-relevant stimulus and the response was observed, suggesting that the context was part of a higher-order binding structure along with the other stimulus and the response (Mayr et al., 2018). This kind of binding that includes more than two elements is referred to as a *configural binding* (Moeller et al., 2016; Qiu et al., 2022a,b). It is assumed that, in this configural binding structure, the context and the task-relevant stimulus form a compound that is bound with the response (Mayr et al., 2018; Qiu et al., 2022b). In addition to configural and binary binding, there are also observations suggesting that the contextual features may not be bound (e.g., Frings and Rothermund, 2017; Qiu et al., 2022b). Unveiling when and how the context is integrated can offer insights into the architecture of bindings between contextual features and responses, thereby shedding light on how environmental information influences human behavior. Therefore, the main purpose of the current study was to elucidate the integration of context in an S-R episode, with a particular focus on the inter-trial variability of the contextual information.

Previous studies have identified several factors that affect the stimulus-response binding and retrieval processes (for a review, see Frings et al., 2020), the one most relevant to the current study is attention (e.g., Hommel, 2005, 2007; Hommel et al., 2014; Zmigrod and Hommel, 2009, 2011; Zmigrod et al., 2009), which was demonstrated to affect feature integration in different ways. For example, Zmigrod and Hommel (2009) manipulated whether the features of auditory stimuli (such as pitch, loudness, and location) were task-relevant or not, which was expected to consequently influence the amount of attention devoted to these features. The results showed a more pronounced after-effect of stimulus-response binding for the task-relevant feature compared to the task-irrelevant one. For another example, Qiu et al. (2022b) manipulated the saliency level of auditory contextual stimuli by altering their loudness and emotional valence. The results showed that the binding structure of context varied with its saliency level: The context of low-saliency was not involved in either kind of binding, the context of moderate-saliency was integrated into a configural binding, whereas the context of high-saliency was directly bound with the response in a binary fashion. Presumably, the increase of saliency level results in more attention being attracted to the contextual stimulus, thereby affecting whether it is perceived as background noise which may not be bound, as a part of a stimulus compound that is bound with the response, or as an distinct “object” akin to the target and the distractor which is directly bound with the response (Moeller et al., 2016; Qiu et al., 2022b).

Apart from task-relevance and saliency, stimulus uncertainty has been discussed as one factor influencing attention (Frings et al., 2019). Following the selection history framework of attentional control (Awh et al., 2012), Frings et al. (2019) posited that the uncertainty in stimulus-related history elicited a cognitive state called “curiosity”, which motivates human beings to reduce uncertainty by exploring it (Berlyne, 1949, 1960) and thus automatically attracts attention. In a previous study by Qiu et al. (2022a), the impact of stimulus uncertainty on context integration was examined by manipulating the inter-trial variability of context. Participants were exposed to either two (low-variability) or eight (high-variability) contextual stimuli throughout the entire experiment. The results revealed that the context in the low-variability condition was integrated into a configural binding, whereas the context in the high-variability condition was bound directly with the response in a binary fashion. These findings echoed the previously described attentional influence on the binding structure of context observed in Qiu et al. (2022b), but they did not clarify the boundary of context integration within the domain of stimulus uncertainty. Therefore, one objective of the current study was to investigate the presence of a variability threshold for integration that determines whether the context is involved in the previously mentioned binding structures or not.

Furthermore, Frings et al. (2019) discussed relative frequency and the number of alternatives as aspects of stimulus uncertainty. It remains unclear whether the transformation of the binding structure (i.e., from configural to binary) observed in Qiu et al. (2022a) was due to the increased number of different contextual stimuli per se, or to the significantly reduced relative frequencies of the context as well. The current study thus was also designed to offer an initial assessment of how these two factors—relative frequency and the number of alternatives—influence the binding structure of context.

### The current study

**Figure 1:**
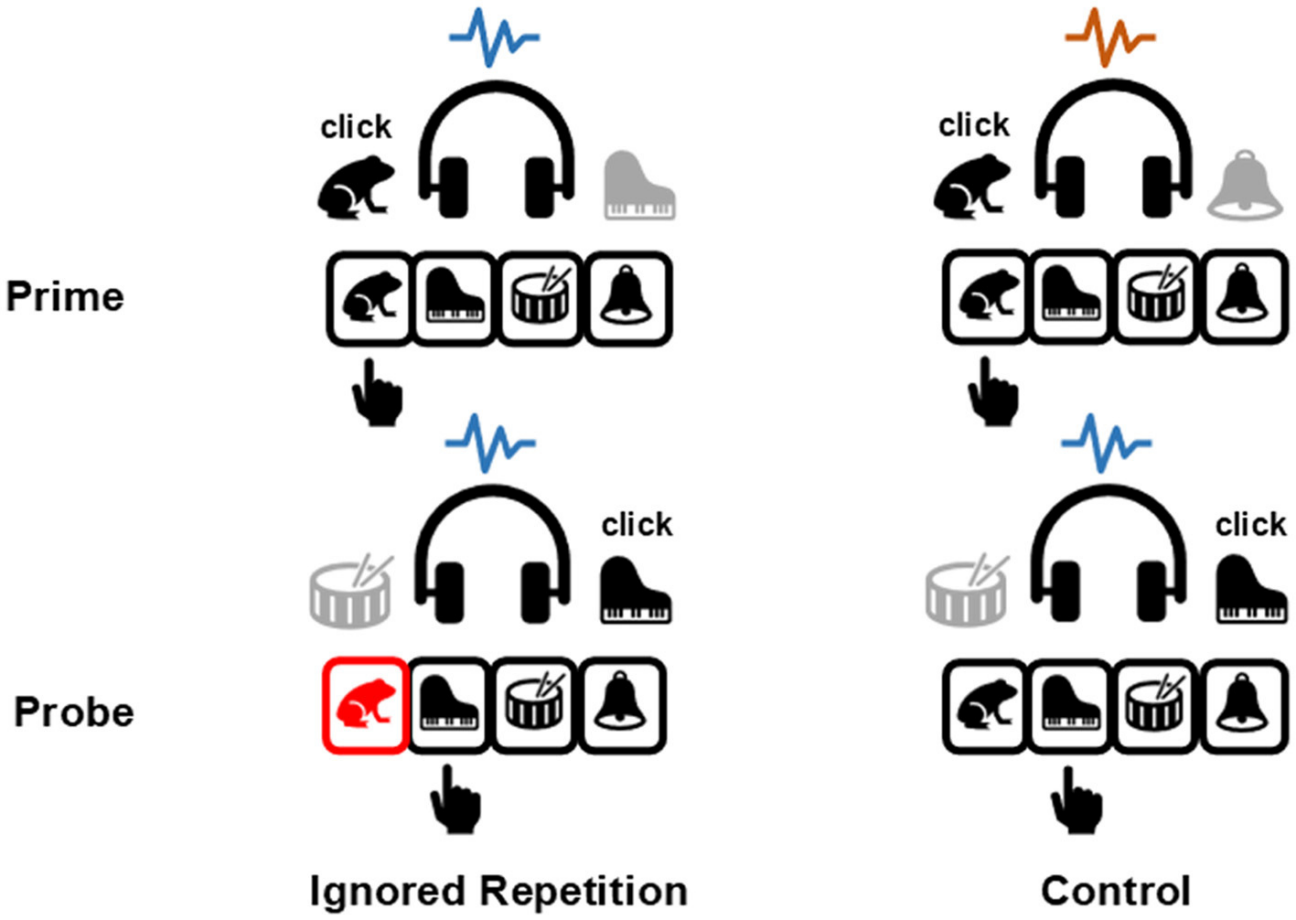
Illustration of the four-alternative negative priming paradigm in the auditory modality. Stimuli in dark are targets which participants should respond to, stimuli in gray are distractors which participants should ignore. The blue and orange wavy lines represent different context tones, which can be repeated or changed between the prime and the probe presentations. In the ignored repetition condition, the distractor stimulus in the prime will function as target in the probe (in this example, the piano sound); whereas no stimulus repetition between the prime and the probe occurs in the control condition. The response key in red indicates an erroneously committed prime response, which is the frog key in this example. The black hand points to the correct response key in each presentation.

The current study employed the auditory four-alternative negative priming paradigm (Mayr and Buchner, 2006) which was also used by Qiu et al. (2022a). As depicted in Figure 1, four environmental sounds served as the target and the distractor stimuli, each was assigned to a specific response key. Participants' task was to identify the target sound via an appropriate response key, and to ignore the simultaneously displayed distractor sound. In the ignored repetition condition, the prime distractor stimulus served as the target in the probe. However, there was no stimulus repetition between the prime and the probe in the control condition. According to the aforementioned stimulus-response binding and retrieval processes, the prime distractor stimulus and the prime response can be spontaneously integrated into an S-R episode. Upon re-encountering the distractor stimulus in the probe, the established S-R episode is retrieved. Consequently, participants are more likely to incorrectly commit the prime response in the probe in the ignored repetition condition than in the control condition, which was considered one of the reasons for the emergence of the so-called negative priming effect (Mayr and Buchner, 2006), the impaired responding toward a previously ignored stimulus (Tipper, 1985).

The four-alternative forced choice task allows for the separation of the incorrect probe response that repeats the prime response from other types of responses, such as incorrect responses to the probe distractor. The stimulus-response binding and retrieval processes induced by the repetition of the distractor stimulus could thus be examined by comparing the prime response errors between the ignored repetition and the control conditions. To this end, Mayr and Buchner (2006) utilized a multinomial processing tree (MPT) model (for a detailed description of the model, please see the Method section) to analyze probe response frequencies across different conditions. Based on the probe response frequencies, the MPT model can estimate and compare the conditional probability of incorrectly committing the prime response in the probe under different conditions. The results showed an increased probability of committing errors with the former prime responses when a stimulus was repeated than when it was changed, a phenomenon termed the *prime-response retrieval effect* thereafter. This effect was documented as a clear indicator of a binary binding between a stimulus and the response (Frings et al., 2015; Mayr et al., 2018; Qiu et al., 2022a,b, 2023). When examining context integration, on the one hand, a significant prime-response retrieval effect caused by the sole repetition of the contextual stimulus is taken as evidence for a binary binding between the context and the response. On the other hand, a larger prime-response retrieval effect induced by the repetition of the prime distractor stimulus in the context repetition condition, as compared with the context change condition, is considered evidence for a configural binding among the context, the distractor, and the response (Mayr et al., 2018; Qiu et al., 2022a,b).

The contextual stimuli used by Qiu et al. (2022a) were adopted in the current study, but the inter-trial variability of context was manipulated to range from the minimum to the maximum degree across five experimental groups. Specifically, in the minimum-variability group, the presentation pattern of two context tones was consistent within each block (please see the Method section for a detailed description regarding the presentation pattern of context in each group). In the low-variability group, the two context tones were presented in a fixed, predictable order. The group with a moderate level of variability was comparable to the aforementioned low-variability condition in Qiu et al. (2022a), where the two context tones were presented in a random order. In the high-variability group, all eight contextual stimuli from Qiu et al. (2022a) were introduced, but with two context tones presented most frequently. Finally, the group with the maximum level of variability was comparable to the aforementioned high-variability condition in Qiu et al. (2022a), where the eight contextual stimuli were presented in a uniformly random order.

**Figure 2:**
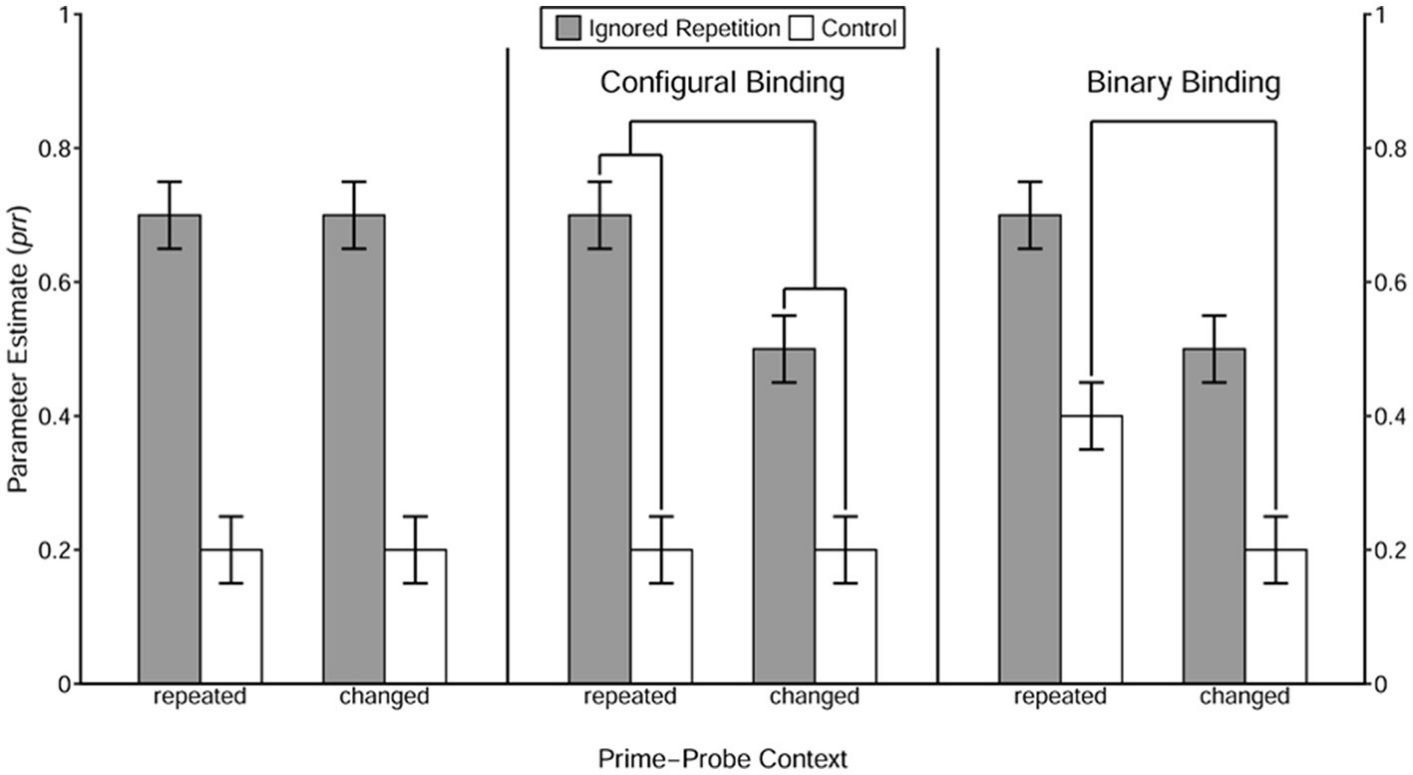
Illustration of prototypical result patterns for each type of context integration. This figure was taken from Qiu et al. (2022b). The value of *prr* parameters reflects the conditional probability of retrieval of the prime response triggered by the repetition of the distractor stimulus and/or the context, which is estimated by the MPT model. The right pattern represents a situation where the repetition of the context itself induces the prime-response retrieval process, suggesting that the context is involved in a binary binding with the response. The middle pattern shows a case where the repetition of the context alone does not enhance retrieval of the prime response, but boosts the prime-response retrieval process induced by repetition of the distractor, indicating that the context is involved in a configural binding with the prime distractor stimulus and the response. The left pattern illustrates a scenario where the retrieval of the prime response is unaffected by the repetition of the context alone or the combination of the distractor and the context, suggesting that the context is not involved in either a binary or a configural binding structure.

The integration of context was expected to vary as a function of its variability level. If an integration threshold for context variability exists, context with the minimum variability may not surpass this threshold. Consequently, repeating the context should neither directly retrieve the prime response nor enhance the prime-response retrieval processes induced by the repetition of the prime distractor stimulus. These observations would suggest that the context is not involved in either the binary binding or the configural binding (see Figure 2 for an illustration of prototypical result patterns of each type of context integration). With a slight increase in variability, the context may exceed the previously described integration threshold, and become involved in a configural binding. Otherwise, the context may not be integrated. For context with variability level identical to that in the low-variability condition in Qiu et al. (2022a), the pattern of results observed in the previous study (i.e., a configural binding) should be replicated. When introducing more contextual stimuli but only slightly changing the relative frequency (considering the two context tones that appeared most frequently), the context may enter into a binary binding with the response. In this case, the number of alternatives may be considered a determinant of the binding structure of context in an S-R episode. Otherwise, the number of alternatives per se cannot determine the binding structure that the context is involved in, suggesting that relative frequency plays a crucial role as well. Finally, context with a variability level identical to that in the high-variability condition in Qiu et al. (2022a) should be directly bound with the prime response in a binary fashion, as observed in the previous study.

## Method

### Participants

Two hundred and fifty-eight participants with normal hearing took part in the experiment for course credit or a 40 RMB monetary reward. 160 of these participants are students at Hunan University of Chinese Medicine. The remaining 98 participants were recruited using NAODAO (https://www.naodao.com/). Data sets of 18 participants had to be excluded due to excessive error rates (> 0.50, as compared with an average of 0.16) in several experimental conditions, suggesting either inability or unwillingness to follow the instructions. The resulting sample consisted of 240 participants (111 females), ranging in age from 18 to 40 years (*M* = 23.03, *SD* = 5.63). This study was approved by the ethics committee of the First Affiliated Hospital of Hunan University of Chinese Medicine, and was conducted in accordance with the 1964 Declaration of Helsinki.

### Materials

The four 300-ms environmental sounds (frog, piano, drum and bicycle bell) used in Qiu et al. (2022a) were employed as target and distractor stimuli in the current study. The loudness level of each sound was approximately 71 dB(A) SPL (sound pressure level). In each presentation, a 20-ms click sound (cue) was played first either to the right ear or to the left ear, indicating the side of the to-be-attended sound (target). The to-be-ignored sound (distractor) would be played simultaneously on the opposite side. Participants' task was to identify the target sound by pressing the appropriate key and to ignore the distractor sound. Four common letter keys *F, V, J, N* were used as response keys, assigned to the sounds of “frog”, “piano”, “drum” and “bicycle bell”, respectively. Participants were instructed to use their index and middle fingers of both hands to press the four keys.

The sine tones that served as context in Qiu et al. (2022a) were employed as well. They were presented alongside the target and distractor sound pairs, and were played simultaneously to both ears to create an impression of originating from a central location. The frequencies of these context tones were (1) 150 Hz, (2) 300 Hz, (3) 400 Hz, (4) 500 Hz, (5), 600 Hz, (6) 700 Hz, (7) 800 Hz, and (8) 900 Hz. Each context tone lasted for 300 ms (including 10-ms attack and decay intervals) and was approximately equal in loudness as the target and the distractor sounds. When added to the sound pair presentation, the context tones only slightly increased the overall loudness (< 3 dB(A) SPL).

Twelve context tone pairs were generated, with the restriction that the frequencies of the context tones within each pair had either even or odd labels [e.g., (1) 150 Hz and (3) 400 Hz, (4) 500 Hz and (8) 900 Hz]. With a frequency difference of at least 200 Hz, the context tones within each pair were readily discriminable from each other. Among the twelve pairs, each of the eight context tones appeared three times.

Each trial was comprised of a prime presentation and a probe presentation. In *ignored repetition* trials, three out of the four sounds were selected as target and distractor in the prime and the probe presentations, with the probe target identical to the prime distractor (see Table 1 for an example). Replacing the prime distractor of each ignored repetition trial with the remaining fourth sound generated the so-called *control* trial. In order to avoid participants anticipating no response repetition between the prime and the probe, *attended repetition* trials and their parallel *attended repetition control* trials were added. The former were generated by selecting three out of the four sounds as target and distractor in the prime and the probe presentations, but with the probe target identical to the prime target. The latter were created by replacing the prime target with the remaining fourth sound. Since no hypothesis was made for attended repetition trials and their parallel control trials, their results will not be reported in the manuscript, but the data were uploaded to PsychArchives for those who are interested. Furthermore, it should be noted that under the described trial generation procedure, each control trial would appear twice: once as a parallel control for an ignored repetition trial and once for an attended repetition trial. To prevent this potential confound, the ignored and attended repetition trials, along with their respective parallel control trials, were systematically divided into two distinct sets (i.e., Set 1 and Set 2). Within each set, control trials were not repeated. Participants were randomly assigned to either Set 1 or Set 2. For more details on the set structure, please refer to Mayr and Buchner (2006).

**Table 1:** Example of stimulus configurations of different trial types in the current study.

	**Ignored repetition**		**Control**		**Attended Repetition**		**Attended Repetition Control**	
	Attended ear	Ignored ear	Attended ear	Ignored ear	Attended ear	Ignored ear	Attended ear	Ignored ear
Prime	Frog	Piano	Frog	Bell	Piano	Bell	Frog	Bell
Probe	Piano	Drum	Piano	Drum	Piano	Drum	Piano	Drum

In this example, the Control trial is identical to the Attended Repetition Control trial. Therefore, the Ignored Repetition and Control trials would have been assigned to one trial set, whereas the Attended Repetition and Attended Repetition Control trials would have been assigned to a separate trial set.

The basic set of experimental trials comprised 48 trials, with 12 trials for each of the four trial types described above. The context tone could be repeated or changed between the prime and the probe presentations, resulting in four different combinations for every pair of context tones [e.g., for the 300 Hz and 700 Hz context tone pair, the combinations are: (1) 300Hz prime context with 300 Hz probe context; (2) 300 Hz prime context with 700 Hz probe context; (3) 700 Hz prime context with 700 Hz probe context; (4) 700 Hz prime context with 300 Hz probe context]. The basic set of experimental trials was implemented for eight times (in general, four times for the context repetition condition, like in Combinations 1 and 3; four times for the context change condition, like in Combinations 2 and 4). The resulting 384 trials were presented in a random sequence, and were divided into eight blocks. Note that in each trial, the side for the prime target display was randomly decided, whereas the probe target was consistently presented on the opposite side. This manipulation was intended to prevent identity-location feature mismatches in the ignored repetition condition, which was reported as one potential reason for the emergence of the negative priming effect (Park and Kanwisher, 1994).

**Figure 3:**
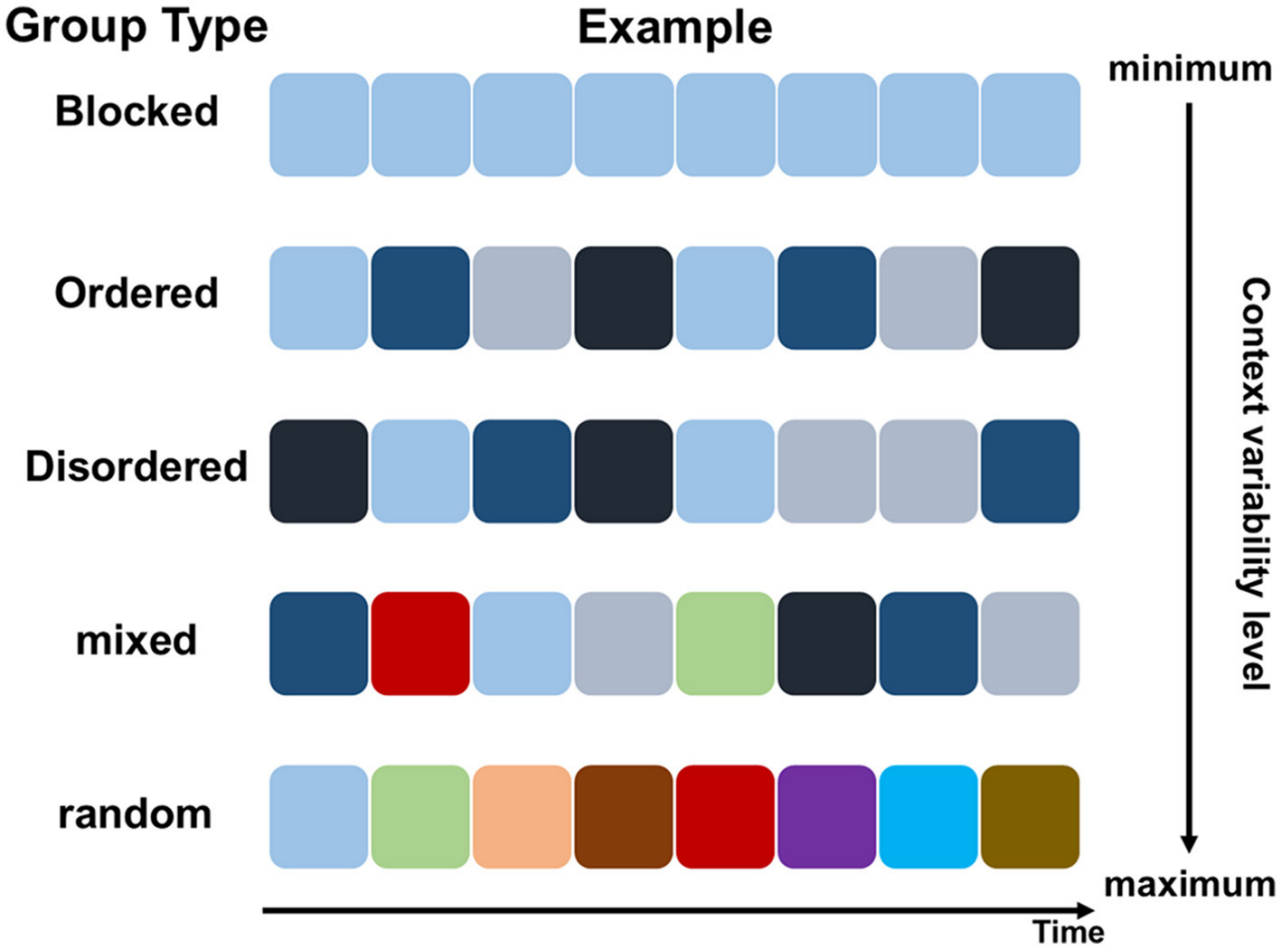
Illustration of context presentation in different experimental groups in the current study. The example shows context of eight trials in sequence within one block for each Group Type. Different prime-probe context tone combinations are represented by different colored squares. Note that the squares in light blue, dark blue, gray blue, and black blue represent the four combinations of two context tones belonging to one context tone pair.

As mentioned in the Introduction, there were five experimental groups in the current study, each characterized by a different level of inter-trial variability of context, spanning from the minimum to the maximum degree (see Figure 3). The first group used one randomly chosen context tone pair from the previously described 12 pairs. In each block of 48 trials, a specific combination of context tones was presented consistently. The first group was thus referred to as the *blocked* group. The randomly selected context tone pair was also used in the second group and the third group. The second group presented the four combinations of context tones in a fixed order (the *ordered* group hereafter); whereas the third group presented them in a random order (the *disordered* group hereafter). All of the 12 pairs of context tones were used in the fifth group, and all possible combinations were presented randomly. The fifth group was thus referred to as the *random* group. As for the fourth group, it was generated based on the disordered group and the random group, with 75% of the trials taken from the disordered group and the remaining 25% taken from the random group. The fourth group was thus referred to as the *mixed* group.

### Procedure

The experiment was programmed using Psychopy3 (Peirce et al., 2019), and was hosted on the previously mentioned NAODAO platform (which requires converting the Psychopy code into PsychoJS code to run in a browser). Participants received an invitation link that directed them to the experiment. They were first instructed to use headphones and to set the volume to a comfortable level. After familiarizing themselves with the four sounds, participants were introduced to the identification task. There were three training sessions. In the first session, participants learned about the target and distractor sound pairs without context tones. They were instructed to identify the target sound via an appropriate keypress and to ignore the simultaneously presented distractor sound. In the second session, the sound pairs were presented with context tones. Participants were informed that the context tones were task-irrelevant and that they should focus merely on the task itself. Participants had to respond correctly in at least 42% of 12 trials to pass the first and the second training session. Finally, participants experienced 10 experimental trials with prime and probe presentations.

Each experimental trial started with a 20-ms prime cue, which indicated the side where the prime target sound would be displayed. After a 500-ms interval, the prime target and distractor sound pair was presented. The prime response was followed by a 500-ms prime-probe interval, after which the probe cue was displayed on the opposite side to the prime cue. After another 500-ms interval, the probe target and distractor sound pair was displayed. Participants received audio-visual feedback about the accuracy of the prime and the probe responses after each experimental trial. The inter-trial interval was 1,200 ms. Note that responses faster than 100 ms or slower than 3,000 ms were considered as invalid and were not included in the analysis. Participants received warning messages for these responses. As mentioned earlier, the experiment comprised eight blocks with 48 trials in each block (i.e., 384 trials in total). Breaks were offered every 24 trials. Participants could either take a rest or start the next part by pressing the space key. The whole experiment lasted for ~60 min.

### Design and analysis

The experiment comprised a 2 × 2 × 5 mixed design with Trial Type (ignored repetition vs. control), Context Relation (repeated vs. changed) as the within-subject variables, and Group Type (blocked vs. ordered vs. disordered vs. mixed vs. random) as the between-subject variable. The Context Relation factor denotes whether the context tone was repeated or changed between the prime and the probe presentations. It should be noted that the Context Relation factor varied from trial to trial in all experimental groups, except in the blocked group, where the Context Relation remained consistent within each block. As for dependent variables, apart from averaged probe reaction times and overall probe error rates, probe response frequencies were analyzed as the primary dependent variable of interest in the current study.

G^*^Power program (Faul et al., 2009) was used for sample size calculation in the current study. To ensure sufficient statistical power to observe the basic negative priming effect, sample size was calculated with the purpose to detect a small-sized effect (i.e., *f* = 0.20, as defined by Cohen, 1988) of context variability on the contextual modulation of the negative priming effect. Given the desired levels of α = β = 0.05, and an assumed correlation of ρ =0.2 (taken from Qiu et al., 2022a), data had to be collected from 195 participants. The final sample comprised 240 participants, thus the power was slightly larger (1- β = 0.98) than what was originally planned for. Note that P-values of multiple comparisons were reported after Bonferroni-Holm correction (Holm, 1979).

**Figure 4:**
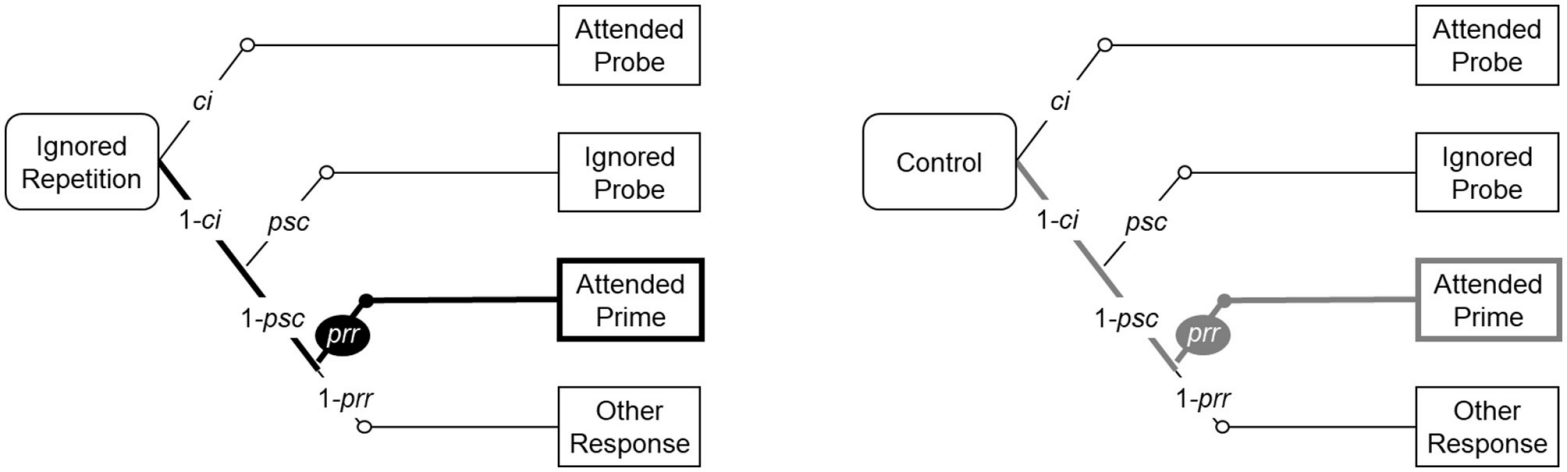
The baseline model for analyzing probe response frequencies. The figure was taken from Qiu et al. (2022a,b). The baseline model comprises two MPT “trees”, one for the ignored repetition condition, one for the parallel control condition. The four “branches” of each “tree” represent the four probe response categories. The label of each “branch” indicates the stimulus that is responded to (e.g., “Attended Probe” is the probe target stimulus, thus this “branch” represents correct responses).

The MPT model (see Figure 4) introduced by Mayr and Buchner (2006) was implemented in the current study to estimate and compare the probability of prime-response retrieval processes for different experimental conditions (Please see Hu and Batchelder, 1994 for a detailed description regarding MPT modeling). This specific model is referred to as the baseline model hereafter. The baseline model describes four different probe response categories in the ignored repetition condition (represented by the abbreviation “IR” in the following) and the control condition (represented by the abbreviation “C”), respectively: (1) correct response (i.e., correct identification, with probability *ci*); (2) incorrect response to the probe distractor (i.e., probe stimulus confusion, with probability *psc*); (3) incorrect execution of the prime response (i.e., prime-response retrieval, with probability *prr*); (4) incorrect response with the remaining fourth response option.

To test the integration of the prime distractor stimulus and the response, a restriction of the equivalence of the *prr* parameters between the ignored repetition condition and the control condition (i.e., *prr*_IR_ = *prr*_C_) should be added to the baseline model. A significant misfit between the restricted model and the empirical data would indicate for the prime-response retrieval effect induced by the repetition of the prime distractor stimulus, suggesting a binary binding between the prime distractor stimulus and the response.

To test the integration of the context, the baseline models for the ignored repetition condition and the control condition should be combined into a joint model for each experimental group. The binary binding between the context and the response was investigated first, by adding a restriction of the equivalence of the *prr*_*C*_ parameters between the context repetition and the context change conditions. If the restricted model significantly misfits the empirical data, then the prime-response retrieval effect induced by the repetition of the context *per se* is significant, which is considered evidence for a binary binding between the context and the response.

Then, the configural binding among the context, the prime distractor stimulus, and the response was investigated. The analysis corresponds to an interaction analysis between Trial Type and Context Relation. Such an interaction in MPT modeling analysis requires reparameterization of the joint model (Please see the Appendix of Qiu et al., 2022b for a detailed description of the reparametrized model and the interaction analysis used in the current study; please see Knapp and Batchelder, 2004 for the reparameterization methods). In the reparametrized model, the prime-response retrieval effect induced by the repetition of the prime distractor stimulus is represented as the difference of the *prr* parameters between the ignored repetition condition and the control condition (i.e., *prr*_IR_ – *prr*_C_). If equating (*prr*_IR_ – *prr*_C_) across the context repetition condition and the context change condition results in a significant misfit between the restricted model and the empirical data, this would indicate contextual involvement in a configural binding, consistent with the findings by Mayr et al. (2018), Qiu et al. (2022a), Qiu et al. (2022b). The model analysis described above was conducted using the multiTree software (Moshagen, 2010).

## Results

### Reaction times and error rates

A 2 (Trial Type: ignored repetition vs. control) × 2 (Context Relation: repeated vs. changed) × 5 (Group Type: blocked vs. ordered vs. disordered vs. mixed vs. random) mixed models ANOVA was applied to probe reaction times and error rates. The results showed a significant main effect of Trial Type in reaction times, *F*_(1, 235)_ = 86.05, *p* < 0.001, $\eta_{\text{p}}^{2}$ = 0.27, and in error rates, *F*_(1, 235)_ = 83.64, *p* < 0.001, $\eta_{\text{p}}^{2}$ = 0.26. Probe responses were slower (*M*_difference_ = 49 ms) and more error prone (*M*_difference_ = 0.03) in the ignored repetition compared to the control condition, which revealed a significant negative priming effect. There was also a significant main effect of Group Type in reaction times, *F*_(4, 235)_ = 8.64, *p* < 0.001, $\eta_{\text{p}}^{2}$ = 0.13. Probe responses were slower in the blocked group than in the ordered group (*M*_difference_ = 195 ms) and disordered group (*M*_difference_ = 154 ms), both *ts* > 3.46, *ps* < 0.01. Probe responses were also slower in the mixed group than in the ordered group (*M*_difference_ = 209 ms) and the disordered group (*M*_difference_ = 168 ms), both *ts* > 3.79, *ps* < 0.01. Unintentionally, participants who took part in the experiment for course credit were predominantly assigned to the blocked and the mixed groups by the program. The longer reaction times observed in these two groups may reflect certain response tendencies of these participants, such as being more cautious when pressing the keys. However, both groups did not exhibit significantly lower error rates compared to the others, providing no evidence of a speed-accuracy tradeoff strategy^1^. Finally, except for a significant main effect of Context Relation in reaction times (*M*_difference_ = 9 ms), *F*_(1, 235)_ = 4.42, *p* < 0.05, $\eta_{\text{p}}^{2}$ = 0.02, none of the other main or interaction effect was significant, all *Fs* < 1.78, *ps* > 0.13.

### MPT model results

**Figure 5:**
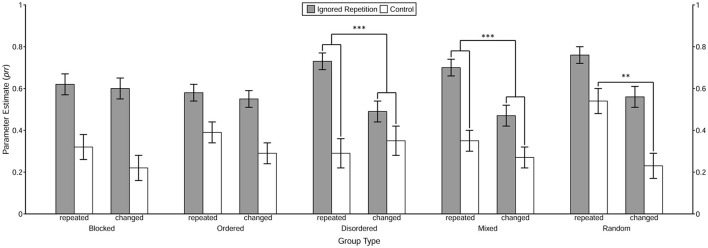
Probability estimates for the model parameters representing the probability of prime-response retrieval (prr) as a function of Trial Type, Context Relation, and Group Type. The value of *prr* parameters reflects the conditional probability of retrieval of the prime response triggered by the repetition of the distractor stimulus and/or the context, which is estimated by the MPT model. The error bars depict the standard errors of the means. Annotation shows significant comparisons indicating configural binding of the context in the disordered and the mixed group, as well as binary binding of the context in the random group. The symbols “***” and “**” indicate *p* < 0.001 and *p* < 0.01, respectively.

The prime-response retrieval effect induced by the repetition of the prime distractor stimulus in each of the 2 (Context Relation) × 5 (Group Type) conditions was tested first. With the restriction *prr*_IR_ = *prr*_C_, the goodness-of-fit test showed significant misfits in most conditions, *G*^2^*s* > 7.42, *ps* < 0.01, ωs > 0.04, except for the context change condition in the disordered group, *G*^2^(1) = 2.57, *p* = 0.11, ω = 0.03. These results clearly demonstrate the prime-response retrieval process induced by the repeated prime distractor stimulus under both the context repetition and the context change conditions in the blocked, ordered, mixed, and random groups, as well as the context repetition condition in the disordered group. The estimated value of the *prr* parameters in each 2 (Trial Type) × 2 (Context Relation) × 5 (Group Type) condition is presented in Figure 5.

Then, the prime-response retrieval effect induced by the repetition of the context *per se* was tested for each of the experimental groups. With the restriction of equivalence of the *prr*_C_ parameters between the Context Relation conditions, the goodness-of-fit test showed a significant misfit only in the random group, *G*^2^(1) = 10.20, *p* < 0.01, ω = 0.04, but not in the remaining four groups, all *G*^2^*s* < 1.86, *ps* > 0.17, ωs < 0.02. These results indicate a binary binding between the context and the prime response in the random group.

Finally, the contextual modulation of the prime-response retrieval effect induced by the repeated prime distractor stimulus was examined using interaction analysis in MPT modeling. The analysis showed that in both the disordered and the mixed groups^2^, the prime-response retrieval effect induced by the repetition of the prime distractor stimulus was larger in the context repetition condition than in the context change condition, both *G*^2^*s* > 12.64, *ps* < 0.001, ωs > 0.04, indicating a configural binding among the prime distractor stimulus, the context and the prime response. As for the remaining three groups, the goodness-of-fit test did not show any significant misfit, all *G*^2^*s* < 0.85, *ps* > 0.35, ωs < 0.02.

## Discussion

In the current study, the influence of stimulus uncertainty on the integration of context within an S-R episode was investigated, with a focus on the inter-trial variability of the contextual stimulus. The context variability was manipulated to range from the minimum degree to the maximum degree across five experimental groups. Specifically, in the blocked group, the presentation pattern of two context tones was consistent within each block. In the ordered group, the two context tones were presented in a fixed order, but were presented in a random order in the disordered group. In the mixed group, all eight context tones were presented randomly, but with two of them occurring most frequently. In the random group, the eight context tones were presented in a uniformly random order.

The current results revealed that the context was not involved in either a binary or a configural binding in the blocked and the ordered groups^3^. However, a pattern of configural binding was observed in the disordered group, aligning with the previous findings of Qiu et al. (2022a). Collectively, given the increased levels of variability across these three groups, it is reasonable to infer that there exists an integration threshold for context variability that determines whether the context is involved in the previously described binding structures or not. In the mixed group, the current results did not show a pattern of a binary binding between the context and the response. Instead, the context was involved in a configural binding with the prime distractor stimulus and the response, similar to the disordered group. As expected, the context in the random group was observed to be bound directly with the response in a binary fashion, replicating the previous findings of Qiu et al. (2022a). Combining observations from the disordered, the mixed, and the random group, the current findings suggest that merely increasing the number of alternatives is insufficient for the context to be bound in a binary fashion with the response; a combination with a significantly reduced relative frequency is required. Taken together, findings of the current study reveal that the inter-trial variability is one determinant of the integration of context in an S-R episode.

The observation of a variability threshold for context integration corroborated and enriched the assumptions about the binding principle of saliency by Hommel (2004). Following this notion, a stimulus is integrated only if its saliency level exceeds the threshold required for integration; otherwise the stimulus may not be bound (e.g., Dutzi and Hommel, 2009; Qiu et al., 2022b). The current study provides empirical support for an extension of the binding principle of saliency into the realm of stimulus variability, showing that the inter-trial variability of context determines whether the contextual information will be integrated into a previously described configural/binary binding structure or not. Moreover, the current findings also support and extend the assumption of a second saliency threshold proposed by Qiu et al. (2022b), which determines the specific binding structure that a stimulus is involved in. Specifically, if the saliency of a stimulus is sufficient for integration but does not meet the second threshold, the stimulus will be involved in a configural binding. Otherwise, the stimulus will be bound in a binary fashion with the response. Incorporating these perspectives into the current study suggests that there may be a second threshold for stimulus variability as well, which determines whether the stimulus enters into a configural binding or a binary binding. Collectively, the current study provides a comprehensive description of how the inter-trial variability of context affects its integration in an S-R episode, thereby extending the previous theoretical concepts of saliency thresholds.

The differences in how the context is integrated across various levels of inter-trial variability may result from differences in attention allocated to the contextual stimulus (Chao, 2009), presumably due to changes in stimulus uncertainty in the selection history (Frings et al., 2019). Given that attention has been shown to affect sensory inputs and representations (e.g., Kizilirmak et al., 2022; Mehrpour et al., 2020), stimulus uncertainty may influence the perception of the contextual stimulus in a similar way as stimulus saliency (Qiu et al., 2022b). Specifically, a completely predictable contextual stimulus may be dismissed as meaningless background noise and not integrated into either a configural binding or a binary binding structure, as observed in the blocked and the ordered groups in the current study. As variability increases, the contextual stimulus may be perceived as background of the task-relevant stimuli (Frings and Rothermund, 2017; Qiu et al., 2022a,b). Consequently, the context forms a compound with the prime distractor stimulus, and the compound becomes bound with the prime response, resulting in the configural binding observed in the disordered and the mixed groups. Finally, when variability is sufficiently high, the contextual stimulus is more likely to be perceived as an individual object similar to the target and the distractor (Frings and Rothermund, 2017; Qiu et al., 2022a,b), and thus enters into a binary binding with the prime response. This is exactly what was observed in the random group of the current study. It may be of interest for future studies to further investigate the influence of inter-trial variability on stimulus perception across a more elaborate spectrum, which could elucidate the specific transformation of stimulus representation. With that being said, it should be noted that the perception-related mechanism is one potential reason for the influence of variability. Future studies will be required to delve deeper into the mechanisms underlying the current observations.

Frings et al. (2019) identified relative frequency and the number of alternatives in the selection history as aspects of stimulus uncertainty. Given the patterns of configural binding observed in the disordered and the mixed group, as well as the pattern of binary binding shown in the random group, the current study suggests that both relative frequency and the number of alternatives contribute to the transformation from a configural binding structure to a binary binding structure. However, these two aspects alone do not seem to explain very well the difference in context integration between the ordered and the disordered groups. Generally speaking, the contextual stimuli in these two groups are comparable not only in relative frequency but also in the number of alternatives. Nevertheless, the context in the disordered group was involved in a configural binding, whereas the context in the ordered group was not.

Presumably, apart from relative frequency and the number of alternatives, unpredictability, which reflects the difficulty in accurately anticipating the timing or the occurrence of stimuli within a sequence (e.g., Koppe et al., 2015; Tsogli et al., 2022), also contributes to stimulus uncertainty. In the current study, the contextual stimuli in the ordered group were presented in a fixed sequence; whereas the contextual stimuli in the disordered group were presented randomly. The level of unpredictability is thus expected to increase from the ordered group to the disordered group, which may increase stimulus uncertainty. Therefore, more attention was automatically attracted to the contextual stimulus in the disordered group (Frings et al., 2019), thereby influencing context integration in S-R episodes. In light of these findings, future studies are required to comprehensively delineate the influence of different aspects of stimulus uncertainty (including unpredictability, relative frequency, and the number of alternatives) as well as the interplays among them on the stimulus-response binding and retrieval processes.

Finally, it should be noted that while the presence of a retrieval effect (e.g., the prime-response retrieval effect in the current study) provides strong evidence for integration, the absence of such retrieval effect does not necessarily imply that the binding process did not occur (for a recent overview of binding and retrieval control, please see Hommel, 2022). On the one hand, several factors could potentially hinder retrieval without affecting binding. Following this notion, the context in the current blocked and ordered groups might be integrated in some way, but the retrieval process was somehow impaired, leading to the result pattern of no retrieval effect triggered by the context. On the other hand, the strength of the binding itself may influence retrieval as well. Even if the binding of contextual stimuli has occurred, it may not be strong enough to produce a detectable retrieval effect. Moreover, there may also be binding structures that are challenging to detect via the method used in the current study. In light of these considerations, the absence of a prime-response retrieval effect under certain conditions of the current study (i.e., the blocked and the ordered groups) should be merely taken as tentative evidence that context was not integrated into an S-R episode. Future research adopting alternative methods of assessing binding, such as neuroimaging techniques or electrophysiological measures, could provide further insights into the underlying processes.

To sum up, the current study investigated the integration of context in an S-R episode by manipulating the inter-trial variability of the contextual stimulus across a spectrum. Results showed that the context with the minimum to low variability did not exhibit a pattern of integration; the context with moderate to high level of variability was involved in a configural binding with the prime distractor stimulus and the prime response; whereas the context with the maximum variability was bound directly with the response in a binary fashion. Taken together, the current study indicates that the inter-trial variability of context is one determinant of the integration of context in an S-R episode, thereby shedding light on the influence of different environmental information on human behavior.

## Data Availability

The datasets presented in the current study are available on https://doi.org/10.23668/psycharchives.15531.

## Footnotes

^1^As mentioned in the Design and Analysis section, the probe response frequencies were analyzed as the primary dependent variable of interest in the current study (the results are reported in the MPT model results section). Based on the frequencies, the MPT model analysis was employed to estimate and compare the conditional probabilities of different probe responses. The most relevant probability for the current purpose is that of incorrectly committing the prime response in the probe. Although we acknowledge that the participants' motivations (for course credit or for monetary reward) may have affected their performance to some extent, there is no compelling reason to believe that this difference would specifically impact the probability of committing particular probe responses, which constitutes the main findings of the current study.

^2^All trials in the mixed group were included in the model analysis. Please note that when removing trials with less frequently presented contexts, the model analysis yielded a similar result pattern, i.e., a configural binding among the context, the distractor, and the response, *G*^2^(1) = 12.02, *p* < 0.001, ω = 0.05.

^3^As mentioned in the Design and Analysis section, the Context Relation factor was manipulated differently between the blocked and ordered groups (i.e., varying from block to block in the blocked group and from trial to trial in the ordered group). Despite this difference, both groups showed similar result patterns of MPT model analysis, indicating that the inter-group difference of the Context Relation factor may not significantly affect participants' behavior in the current study.
